# Supplementary material for: The genome sequence of the mottled worm, Allolobophora icterica (formerly Aporrectodea icterica ) (Savigny, 1826)
Source: Wellcome Open Res. 2024 Sep 26;9:556. [Version 1] doi: 10.12688/wellcomeopenres.23066.1 (PMC11530748; doi:10.12688/wellcomeopenres.23066.1)
Supplement: Peer review history

# Reviewer report: Reviewer response for version 1

**DOI:** 10.21956/wellcomeopenres.25402.r109049

**Published:** 2025-02-14

**Authors:** Yi-Jyun Luo

**Affiliations:** 1 Biodiversity Research Center Academia Sinica, Taipei City, Taipei City, Taiwan

This paper presents a chromosome-level assembly of the earthworm *Allolobophora icterica*. As with other genome notes from the Darwin Tree of Life Project, the genome was sequenced using their gold-standard approach—PacBio long-read and Hi-C sequencing. The assembly was scaffolded into 16 chromosomal pseudomolecules (n = 16), which is at the lower end compared to other earthworm genomes (n = 17–18), suggesting lineage-specific chromosome fusion.

 This genome assembly is a valuable resource for the community and will contribute to various fields in ecology and evolution. In our recent study on annelid phylogenomics (https://doi.org/10.1093/molbev/msae172), we used the A. icterica genome (formerly Aporrectodea icterica) to show that while Metaphire vulgaris underwent a recent whole-genome duplication (WGD), A. icterica did not. We also found that gene order within earthworms appears relatively stable but shows considerable rearrangement when compared to other annelid species.

 The continued efforts of the Darwin Tree of Life Project in generating high-quality chromosome-level assemblies are commendable and will greatly advance our understanding of genome structure evolution.

 Minor comments:

 1. A recurring issue in genome notes is the lack of clarity on how the haplotype was selected in the "Genome sequence report" section. Providing more details on the selection criteria or methodology would improve transparency for readers.

 2. Another common issue is the omission of information about the tissue used for genome sequencing. Clarifying the tissue type would be valuable, as it may be relevant for researchers sequencing related species.

Are sufficient details of methods and materials provided to allow replication by others?

Partly

Is the rationale for creating the dataset(s) clearly described?

Yes

Are the datasets clearly presented in a useable and accessible format?

Yes

Are the protocols appropriate and is the work technically sound?

Yes

Reviewer Expertise:

Evolutionary Genomics, Evolutionary Developmental Biology

I confirm that I have read this submission and believe that I have an appropriate level of expertise to confirm that it is of an acceptable scientific standard.

## References

[1] : Annelid Comparative Genomics and the Evolution of Massive Lineage-Specific Genome Rearrangement in Bilaterians. *Mol Biol Evol* .2024;41(9) : 10.1093/molbev/msae172 10.1093/molbev/msae172 39141777 PMC11371463

# Reviewer report: Reviewer response for version 1

**DOI:** 10.21956/wellcomeopenres.25402.r109050

**Published:** 2024-11-01

**Authors:** Daniel F Marchán

**Affiliations:** 1 Biodiversity, Ecology and Evolution Department, Faculty of Biology, Complutense University of Madrid, Madrid, Community of Madrid, Spain

The dataset generated is of high quality and highly valuable as a resource for both soil biologists and genetics researchers.

 The presentation is clear and sound, and easy to follow. Accessibility to the data by other researchers should be easy.

 No further comments can be added in other to improve the manuscript.

Are sufficient details of methods and materials provided to allow replication by others?

Yes

Is the rationale for creating the dataset(s) clearly described?

Yes

Are the datasets clearly presented in a useable and accessible format?

Yes

Are the protocols appropriate and is the work technically sound?

Yes

Reviewer Expertise:

Earthworm molecular phylogenetics and evolution

I confirm that I have read this submission and believe that I have an appropriate level of expertise to confirm that it is of an acceptable scientific standard.

# Reviewer report: Reviewer response for version 1

**DOI:** 10.21956/wellcomeopenres.25402.r101862

**Published:** 2024-10-02

**Authors:** Dong Zhang

**Affiliations:** 1 Lanzhou University, Lanzhou, Gansu, China

The authors presented a paper entitled "The Genome Sequence of the Mottled Worm, *Allolobophora icterica *(formerly *Aporrectodea icterica*) (Savigny, 1826)," which primarily describes the sequencing and assembly of a high-quality genome of an individual mottled worm, *Allolobophora icterica*. The manuscript is well-written, and the methods are sound. I recommend accepting this manuscript.

Are sufficient details of methods and materials provided to allow replication by others?

Yes

Is the rationale for creating the dataset(s) clearly described?

Yes

Are the datasets clearly presented in a useable and accessible format?

Yes

Are the protocols appropriate and is the work technically sound?

Yes

Reviewer Expertise:

genomics

I confirm that I have read this submission and believe that I have an appropriate level of expertise to confirm that it is of an acceptable scientific standard.
